# There’s no app for that! perspectives on engaging diverse communities to promote equitable care

**DOI:** 10.1177/20552076251348095

**Published:** 2025-06-04

**Authors:** Jennifer Lane, Brittany Barber, Courtney Pennell, Kris Lane, Megan White, Noah Doucette

**Affiliations:** 1 3688 Faculty of Health, School of Nursing, Dalhousie University, Halifax, NS, Canada; 2 3682 IWK Health Centre, Halifax, NS, Canada; 3 Tajikeimɨk, Millbrook, NS, Canada; 4 103986 College of Physicians and Surgeons of Nova Scotia, Bedford, NS, Canada; 5 Nova Scotia Health, Halifax, NS, Canada

**Keywords:** Health equity, community engagement, health disparities, structural determinants of health

## Abstract

Engaging patients and communities in the development and evaluation of mHealth applications can optimize useability, user adherence, health outcomes, and transparency of how personal health data is used, stored, and transferred to commercial partners. This commentary is informed by an event that aimed to invite knowledge sharers/users from equity-denied groups to provide feedback about a preliminary set of questions meant to collect socio-structural health determinants information and their potential use within mHealth applications. Three key lessons were learned: (1) challenges of reciprocity, (2) concerns of responsible data stewardship, and (3) processes of building trust for meaningful community engagement. Responding to historical and ongoing injustices is critical for building trust and supporting successful uptake of health technologies with communities. Soliciting feedback from community once decisions about implementation have been made may come across as performative or disingenuous, further undermining possibilities to establish and maintain productive relationships that are mutually beneficial to all parties involved. Without concerted effort to improve access to healthcare resources, progress made with mHealth applications may come at the expense of people and communities already underserved within existing healthcare systems.

## Introduction

Mobile health (mHealth) applications – the practice of medicine and public health data supported by mobile devices – have proliferated within healthcare interventions for preventive behaviour change, disease self-management, and real-time monitoring of patient reported health outcomes.^1,2^ mHealth technologies show effectiveness for mental health^3^ and chronic disease^4^ self-management, reduction of hospitalizations and re-admissions,^1^ and surveillance and symptom management of infectious diseases such as COVID-19.^5,6^ Further, mHealth technologies demonstrate promise for reducing healthcare costs, access to healthcare providers,^2^ enhance patient-provider communication,^7^ and improve patient satisfaction.^8^ Despite numerous mHealth benefits, it is unclear the role of patients and communities in supporting the development and evaluation of mHealth applications for optimizing useability and patient adherence, patient health outcomes, and transparency of how personal health data is used, stored, and transferred to commercial partners.^2^

Use of personal health data is a major concern due to the potential for misuse, privacy breach, and discrimination.^9^ Although mHealth enhances availability of detailed individual health data informing patient and provider healthcare decisions, mHealth applications are typically not interoperable with other health information systems^10^ and are limited from inconsistent data standards^9^ and methods for collecting personal health information.^11^ This makes it challenging to monitor data privacy and ownership of big data to promote equitable benefits from precision public health,^12,13^ such as applications of mHealth data to advance our understanding of health determinants across populations.

Impetuses for addressing mHealth data standards (e.g., transparency of data collection methods, use, and data ownership) are essential for improving mHealth application functionality and processes of community engagement, such as utilizing available sociodemographic data to address health disparities and inequitable access to healthcare. This is particularly challenging when limited sociodemographic data is available beyond age, sex/gender, and geographic location.^14^ Further research is needed to explore how patients and communities want to be engaged in strengthening mHealth functionality and their role in co-designing mHealth solutions tailored to individuals and communities that could benefit most.

**Figure 1.**
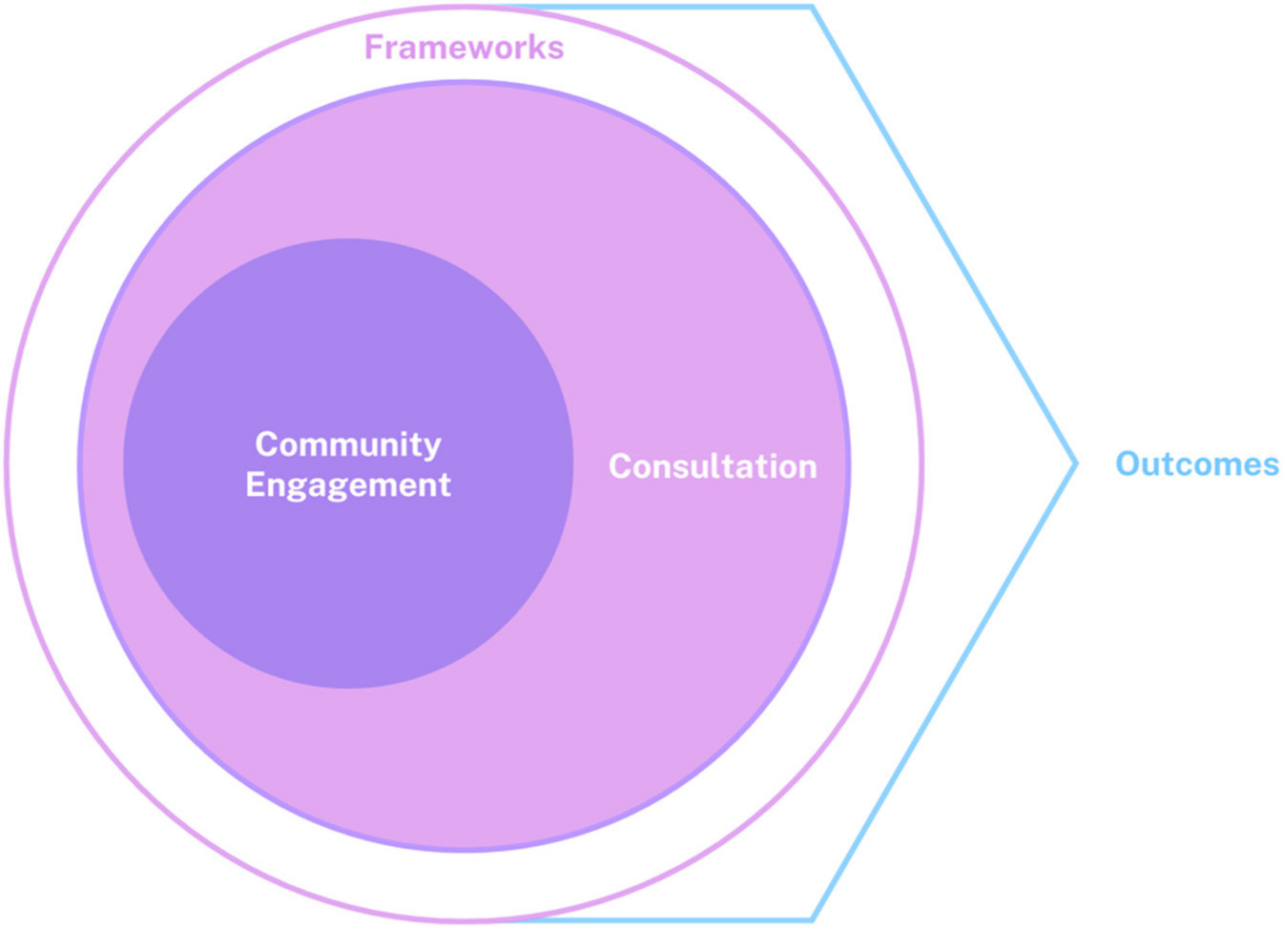
Visual representation of community consultation process.

Purposeful consultation through community engagement within the design and development of mHealth applications has the potential to influence collection and use of health determinants (HD) data, informing where and how to address health disparities across communities where limited HD data exists (see Figure 1 for a visual representation of the authors’ conceptualization of this process). This is particularly important in places with heterogenous populations, rural and urban settings, and regional health authorities. The regional diversity in Nova Scotia (NS), an Atlantic Maritime province in Canada, presents a unique and valuable opportunity to explore health inequities, particularly as new mHealth technologies become available and implemented. This regional diversity includes a large proportion of the population being rurally situated, disproportionate rates of cancer attributable to environmental and industrial pollution (e.g., Black and Mi’kmaq populations),^15^ the highest poverty rate in Canada (increasing by 52%, from 8.6% to 13.1% between 2021 and 2022),^16^ and the highest proportion of gender diverse individuals aged 15 to 34 in Canada.^17^ There is urgent demand to address health disparities and inequitable access to healthcare across NS, such that 11% (or 119,670 people) are without a family doctor^18^ and wait times from referral to treatment have been identified as the highest in Canada (56.7 weeks in NS compared to 21.6 weeks in Ontario).^19^ Although mHealth applications have potential to advance healthcare in NS, consultation through community engagement is a critical first step in understanding acceptability and potential barriers to increasing uptake of mHealth technologies among equity-denied groups. The objective of the present study was to consult people living in NS through community engagement to explore collecting HD data and better understand perceptions of, opinions on, and potential barriers to mHealth implementation.

With an aim to engage knowledge sharers/users from diverse communities, the first and third authors worked together as an independent third party with a health system partner. This arrangement was needed because the third-party partner had pre-existing relationships that the health system partner did not, making the community engagement possible. The community engagement session involved a diverse group comprised of the team who planned and hosted (n = 8) and knowledge sharers/users with whom consultation was being sought (n = 26). Among those represented were Mi’kmaq, African Nova Scotian, Francophone, and 2SLGBTQ community-leaders, decision-makers, health professionals, and youth living in various areas of the province. The community engagement session started with an opportunity for feedback to be offered by the larger group (facilitated by the first author). Smaller groups were formed; virtual and in-person discussions were then facilitated by members of the planning/hosting team. Knowledge sharers/users shared feedback about HD questions as well as community concerns and barriers influencing the implementation of potential mHealth technologies in their communities. Facilitators in each group recorded detailed notes, which were then discussed during a debrief. The first author collated the notes supplied by small-group facilitators, which informed a final list of recommendations and unanticipated barriers. Discussions were not audio- or videorecorded and direct quotations from participants were not included to uphold the integrity of knowledge sharers’/users’ open discussion of sensitive information, privacy, and community understandings of respect. The diverse and equity-denied groups with whom we consulted live with the impacts of historical harm from institutions such as those represented by the researchers, namely higher education and healthcare. More, our research team is attentive to community-specific cultural norms and values. Some members of equity-denied groups, including those on our team and with whom we consulted, feel that audio- or videorecording sensitive community discussions is extractive, intrusive, and would likely have undermined our pre-existing trustful relationships. Given these concerns and contexts, relational trust with communities was prioritized over the accuracy afforded by audio- or videorecording by deeming alternative forms of documentation most appropriate.

## Lessons learned

Three key lessons were learned: 1) challenges of reciprocity, 2) concerns of responsible data stewardship, and 3) processes of building trust for meaningful community engagement.

### Reciprocity

Processes by which benefits are mutually exchanged and shared, including those between researchers, decision-makers, and communities are grounded in *reciprocity*.^20^ A discrepancy between perceived benefits of mHealth applications (by partner organization decision-makers) and unforeseen barriers to their equitable access (experienced by equity-denied groups) was identified during the engagement event, and highlighted the importance of reciprocity when engaging any community, but particularly those who are underserved. The wisdom imparted by members of equity-denied groups that allowed the partner organization to identify unforeseen barriers far surpassed what was received in return. The exchange process lacked in reciprocity because what was shared through community consultation negated perceived benefits of mHealth applications. In other words, the benefits of the mHealth application anticipated by the health system partner (i.e., that community would benefit from the mHealth application) were one-sided in so far as there were unforeseen barriers to implementation. As such, the wisdom imparted by community to the health system partner was received, but little was given in return, which highlights challenges of reciprocity.

During the community feedback session, the implications of implementing mHealth applications for people living in rural parts of NS were discussed. Approximately one fourth of homes and businesses in NS (disproportionately Mi’kmaq communities) do not have adequate telecommunications infrastructure to provide high-speed internet.^21^ Reliable telecom services are crucial to support a wide range of economic and social activities, and vital for implementation of new mHealth applications that aim to improve access to health care. When these barriers were brought to light, decision-making leaders at the event were challenged with addressing concerns, such as increasing efforts to speed up provincial timelines for investing in new telecom services. Without clear timelines of when new technologies would be accessible to equity-denied groups, the implementation of mHealth applications would not be equally available to all people living in NS. Knowledge sharers/users discussed the importance of reciprocity as an essential value and principle, highlighting the discrepancy between lived community experiences (i.e., technological barriers to implementing digital health solutions) and disparities between inequitable outcomes, such as stated benefits of mHealth technologies they would not receive (i.e., promises that beneficial changes were coming).

### Data stewardship

Freely sharing details about why and how data are being collected and used, rights of ownership, and ethical reuse with those from whom the data are being collected is called responsible data stewardship.^22,23^ Concerns around (and the importance of) responsible data stewardship, particularly for equity-denied groups experiencing health disparities in NS, were noted at the community engagement event. Consideration needs to be given to how data will be collected and used, rights of data ownership that require members of underrepresented populations to disclose personal health information to industry partners, and perceived risks of global data monetization markets. The lack of clarity around what regulatory policies guide industry healthcare data stewardship and how personal health information would be protected, especially that which belongs to members of equity-denied groups, resulted in additional concerns that would be particularly relevant when working with industry partners operating outside the region who may be perceived to be exempt from local privacy laws.

### Building trust

Knowledge sharers/users discussed the importance of building trust by addressing embedded racism and other forms of discrimination reinforcing historical inequities via imbalanced power relations. Ongoing barriers to equitable and structurally responsive healthcare in NS have led to disproportionate risk of chronic diseases and worse health outcomes that can span generations for racial and ethnic minority groups, particularly Indigenous and Black communities.^24,25^ History shows that many groups who are currently underserved have also experienced injustices that were directly caused by health researchers, highlighting the need to be intentional about building trust, particularly for those conducting research within or adjacent to health systems.^26,27^

## Insights gained & recommendations

mHealth applications provide opportunities for advancing availability of HD data and means to explore social factors impacting health outcomes however, community engagement can be oversimplified or misunderstood. Responding to historical and ongoing injustices is critical for early stages of building trust and preparing for successful uptake of health technologies with communities. While systems changes are largely performative if they fail to address underlying power structures, they serve as a starting point for meaningful engagement and may reduce the risk of missteps. As such, symbolic efforts in demonstrating respect and building trust can be a step in the right direction to repairing relationships with equity-denied groups *if they are carried out swiftly* and should include addressing issues that may have caused previous missteps to happen.

Organizers do not necessarily have to belong to the community being engaged, but wise practices^28^ suggest that strong relationships are central to authentic consultations with equity-denied groups and that embedding reciprocity as a key guiding principle is critical if community engagement activities are being organized by researchers and health system decision-makers with resources and influence over allocation of healthcare resources to improve access and patient outcomes.^20^ Putting systems of accountability in place to promote reciprocity in decision making and equitable benefit distribution from healthcare investments may decrease discrepancies that work to reinforce health inequities and underservicing in health care. At the least, meaningful and ongoing reflection about who benefits from consulting with community is vital to equitable engagement. Consultation should include clarification around roles and responsibilities, explicit exploration of mutual benefits between parties involved, and transparency around the intent behind engagement, how information is collected, what will be done with it, and expected outcomes. Opportunities to make changes to the consultation process should mirror an informed consent process whereby roles, responsibilities, and other aspects of the planned interactions are discussed in an ongoing manner.

Maintaining a health equity focus when working with a partner organization needs to be top-of-mind or institutional politics may take over, particularly when deciding upon unanticipated next steps. Institutions typically have priorities. Presumably in the case of digital health solutions, the priority is to launch the mHealth platform. If community consultation is not done early and meaningfully through existing relationships and partnerships, then unexpected challenges may arise late in the process and create barriers to the institution moving forward the work that they see as important (or if this moving forward happens, then trust building may be undermined). If feedback from community is what is desired, then late-in-the-process engagement may come across as disingenuous, further undermining possibilities to establish and maintain productive relationships that are mutually beneficial to all parties involved.

Before sensitive personal and health information is gathered, it is crucial to understand the misuse of data belonging to underrepresented communities.^25^ Undeniably, sensitivities will arise when institutions engage in equity work because they are the sites where embedded power structures have the potential for breach of trust, which may be particularly true when there are industry partners involved. These sensitivities will arise on all sides and as such, the focus must remain on equity, or progress for some may inadvertently come at the expense of others. Without exception, the focus must remain on communities if consultation through community engagement is the goal, no matter what sensitivities arise within partner organizations, with industry partners, or anything of the like. Otherwise, institutional priorities may take over, risking the misappropriation of shared knowledge, doing little to address the underlying power relations that cause and perpetuate health inequities, and hindering advancements in health equity.

Health system programs and interventions are commonly piloted with homogenous populations in urban settings, making it challenging to scale across diverse communities, particularly in remote and under resourced settings. The participation of heterogeneous communities is crucial, and engagement strategies should align with community goals and priorities. A broadly inclusive approach was used to engaged individuals who belong to various equity-denied groups with whom there were existing relationships, allowing for a group of individuals with diverse experiences to be involved in the event (e.g., race, sexual orientation, gender). Similarly, it might be advantageous for health system innovations to embed co-design processes that engage communities *from the start* and include strategies for sustaining structurally and culturally safe implementation processes.^29^

Outcomes will vary as they depend on factors influencing community engagement, particularly relational dynamics, such as historical context, existing connections between those involved, and their group membership(s). In the scenario presented, an independent third party was advantageous; the first and third authors could draw on their existing relationships and engage members of various communities in ways that the partner organization could not. Consultation through community engagement included establishing feedback mechanisms to ensure participants had multiple chances to contribute. Follow-up sessions may also be advantageous for keeping community informed and involved (Table 1).

**Table 1.** Process for consultation through community engagement.

Actions	Actors	Example(s)
Through existing relationships, build a team comprised of individuals who belong to diverse groups	Individuals with varying areas of expertise and experience	Lived, professional, clinical, academic, advocacy
Determine diversity within engagement population	Team members with experience in community engagement who wish to involve individuals in their network	General population, geographic distribution, socioeconomic groups
Create an invite list	Team members with experience in community engagement who wish to involve individuals in their network	Diverse representation from various community groups (e.g., Mi’kmaq, African Nova Scotian, Francophone, 2SLGBTQ)
Find a location, set the date, and create a detailed description of the event	Team members who will be involved in organizing the event	Consider accessibility, convenience, and cultural relevance of the location
Create an invitation with general information about the event	Lead researcher and/or designated team member	Use a QR code linking to detailed event information and registration
Facilitate initial feedback session	All participants, with designated lead facilitator from planning/hosting team	Large group discussion to gather initial thoughts and concerns
Organize smaller group discussions	All participants, with designated group facilitators from planning/hosting team	Virtual and in-person discussions to delve deeper into specific topics
Record detailed notes during discussion*	Group facilitators from planning/hosting team	Capture feedback accurately and respectfully by taking notes instead of audio- or videorecording
Debrief and collate notes	Lead researcher and/or designated team member(s)	Summarize key points and identify common themes
Develop final recommendations	Led by lead researcher and/or designated team member(s); team members invited to make suggested revisions	Create a report on collated notes and feedback from all sessions
Share outcomes with participants	Participants	Send to participants and ask for their input on the final report to ensure accuracy and completeness
Revise the report as necessary	Designated team member(s)	Make revisions based on the feedback received to ensure the report reflects participants' input accurately

## Conclusion

Strengthening relationships between equity-denied groups and decision-makers is a foundational part of the intentional and reciprocal process of consultation through community engagement, requiring significant investments of time to align goals and plan for implementing systemic change. Equity-denied groups need to be engaged from the start of project development so that health innovations can be tailored to contexts of community settings, with investments to address underlying health inequities. As a critical first step in improving health system innovations and advancing health equity, insights and key lessons learned from knowledge sharers/users are being shared so they can be used by others trying to build productive relationships and increase decision-maker awareness of how to address cultural, social, and historical inequities.
